# Comprehensive behavioral analysis of ENU-induced Disc1-Q31L and -L100P mutant mice

**DOI:** 10.1186/1756-0500-5-108

**Published:** 2012-02-20

**Authors:** Hirotaka Shoji, Keiko Toyama, Yoshihiro Takamiya, Shigeharu Wakana, Yoichi Gondo, Tsuyoshi Miyakawa

**Affiliations:** 1 Division of Systems Medical Science, Institute for Comprehensive Medical Science, Fujita Health University, Toyoake, Aichi, Japan; 2 Japan Science and Technology Agency (JST), Core Research for Evolutional Science and Technology (CREST), Kawaguchi, Saitama, Japan; 3 National Museum of Emerging Science and Innovation, Koto-ku, Tokyo, Japan; 4 Technology and Development Team for Mouse Phenotype Analysis, Riken BioResource Center, Tsukuba, Ibaraki, Japan; 5 Mutagenesis and Genomics Team, Riken BioResource Center, Tsukuba, Ibaraki, Japan; 6 Center for Genetic Analysis of Behavior, National Institute for Physiological Sciences, Okazaki, Aichi, Japan

## Abstract

**Background:**

*Disrupted-in-Schizophrenia 1 *(*DISC1*) is considered to be a candidate susceptibility gene for psychiatric disorders, including schizophrenia, bipolar disorder, and major depression. A recent study reported that *N*-ethyl-*N*-nitrosourea (ENU)-induced mutations in exon 2 of the mouse *Disc1 *gene, which resulted in the amino acid exchange of Q31L and L100P, caused an increase in depression-like behavior in 31 L mutant mice and schizophrenia-like behavior in 100P mutant mice; thus, these are potential animal models of psychiatric disorders. However, remaining heterozygous mutations that possibly occur in flanking genes other than *Disc1 *itself might induce behavioral abnormalities in the mutant mice. Here, to confirm the effects of Disc1-Q31L and Disc1-L100P mutations on behavioral phenotypes and to investigate the behaviors of the mutant mice in more detail, the mutant lines were backcrossed to C57BL/6JJcl through an additional two generations and the behaviors were analyzed using a comprehensive behavioral test battery.

**Results:**

Contrary to expectations, 31 L mutant mice showed no significant behavioral differences when compared with wild-type control mice in any of the behavioral tests, including the Porsolt forced swim and tail suspension tests, commonly used tests for depression-like behavior. Also, 100P mutant mice exhibited no differences in almost all of the behavioral tests, including the prepulse inhibition test for measuring sensorimotor gating, which is known to be impaired in schizophrenia patients; however, 100P mutant mice showed higher locomotor activity compared with wild-type control mice in the light/dark transition test.

**Conclusions:**

Although these results are partially consistent with the previous study in that there was hyperactivity in 100P mutant mice, the vast majority of the results are inconsistent with those of the previous study; this discrepancy may be explained by differences in the genetic background of the mice, the laboratory environment, experimental protocols, and more. Further behavioral studies under various experimental conditions are necessary to determine whether these *Disc1 *mutant mouse lines are suitable animal models of schizophrenia and major depression.

## Background

Schizophrenia, bipolar disorder, and major depression are common psychiatric disorders with variable phenotypes that typically include hallucinations, delusions, disorganized thinking, anhedonia, decreased motivation, and cognitive deficits in learning, memory and attention. Such psychiatric disorders are well known to be highly heritable, and several genes, such as *DISC1, NRG1*, and *DTNBP1*, have been identified as putative susceptibility genes [1-4]. The *Disrupted-in-Schizophrenia 1 *(*DISC1*) gene on chromosome 1 was originally discovered in a Scottish family carrying a balanced translocation (1, 11)(q42.1;q14.3) that is linked to major psychiatric disorders [e.g., [2,5-8]]. To demonstrate *DISC1 *function and investigate the neurobiological basis of psychiatric disorders, several mouse models have been generated [9-14].

*DISC1 *isoforms are encoded by 13 major exons. The longest one, exon 2, is present in all known splice isoforms and encodes most of the protein head domain. Recently, Clapcote et al. [9] used the method of *N*-ethyl-*N*-nitrosourea (ENU) mutagenesis, which induces point mutations at a high locus-specific rate [15-17], to generate two mouse lines from the progeny of ENU-mutagenized C57BL/6JJcl males and untreated DBA/2JJcl females. Each mouse line has a missense mutation in exon 2 of *Disc1*; mutant transcript *Disc1^Rgsc1393 ^*has a 127A/T transversion that results in the amino acid exchange of Q31L, and mutant transcript *Disc1^Rgsc1390 ^*has a 334 T/C transition that results in the amino acid exchange of L100P in the encoded protein. The behavioral analysis by Clapcote et al. [9] indicated that the mice with mutation Q31L (31 L) showed increased immobility time in the forced swim test, decreased sucrose consumption, reduced social novelty preference in Crawley's social interaction test, deficits in prepulse inhibition, and working memory impairments in the T-maze test. Also, they reported that mice with mutation L100P (100P) displayed hyperactivity in the open field test and deficits in sensorimotor gating in the prepulse inhibition test and working memory in the T-maze test. The study of Clapcote et al. [9] suggested that the two mouse lines with point mutations on the *Disc1 *gene are potential animal models of depression and schizophrenia.

The behavioral phenotypes of mutant mice are strongly influenced by their genetic background, including closely linked genes flanking the target locus, as well as the gene with the mutation of interest. Appropriate controls for genetic background are essential for adequate experimental design and the proper interpretation of data [18-21]. It is estimated that ENU mutagenesis could randomly induce 3, 000 mutations in genomic DNAs in male mice; the mutations would transmit to offspring through the mating of ENU-mutagenized C57BL/6 J male mice with non-treated DBA/2 J female mice [15]. The 31 L and 100P mutant mouse lines could possibly have remaining heterozygous mutations in flanking genes other than *Disc1 *and also contain some residual DBA/2 J genetic background, although they had been backcrossed to C57BL/6 J mice for five generations (N2-N6). Therefore, the behavioral phenotypes of the 31 L and 100P mutant mice might be due to not only the *Disc1 *point mutation, but also to possible confounding genetic factors.

In our laboratory, so far we have assessed the behavioral phenotypes of more than 140 strains of genetically engineered mice and several inbred strains of mice to explore relationships between genes, the brain, and behavior. Our strategy of applying a series of various behavioral tests, which we call a comprehensive behavioral test battery, to the experimentally manipulated mice may contribute to the establishment of animal models of psychiatric disorders, such as schizophrenia and bipolar disorder [22]. Our behavioral test battery is useful for detecting behavioral abnormalities relevant to psychiatric disorders in mutant mice [e.g., [23-28]].

In the present study, to reduce possible confounding genetic factors in *Disc1 *mutant mice, male 31 L and 100P mutant mice were backcrossed to C57BL/6JJcl female mice for two more generations. Thereafter, we subjected the homozygous mutant mice and wild-type control mice to a comprehensive behavioral test battery to precisely evaluate the effects of 31 L and 100P mutations in the *Disc1 *gene on behavioral phenotypes. Neither 31 L nor 100P mutant mice showed significant behavioral differences compared with wild-type control mice in any of the tests for assessing behavioral phenotypes relevant to psychiatric disorders, except that 100P mutant mice showed slightly higher locomotor activity than control mice. The present results did not support the conclusion of Clapcote et al. [9] that the two strains of mutant mice are animal models of depression and schizophrenia.

## Methods

### Generation of Disc1 mutant mice

ENU-mutagenized Disc1-Q31L and Disc1-L100P mutant mouse lines were originally generated by the RIKEN BioResource Center http://www.brc.riken.jp/lab/animal/en/gscmouse.shtml, and were developed by the Toronto Center for Phenogenomics for phenotype testing as described previously [9]. Briefly, by screening the F1 progeny of ENU-mutagenized C57BL/6JJcl males and untreated DBA/2JJcl females, a mouse with the point mutation Q31L or L100P in exon 2 of *Disc1 *was detected. Heterozygous N2 backcross progeny of the founder 31 L heterozygous (DBA/2JJcl × C57BL/6JJcl) F1 males and wild-type C57BL/6JJcl females were backcrossed through the male and female lines to C57BL/6 J for four generations (N3-N6). This line was transferred to the RIKEN BioResource Center, where the homozygous (31 L/31 L, 100P/100P) mutants are being maintained by brother-sister mating. The homozygous male mice were transferred to the National Museum of Emerging Science and Innovation, and were backcrossed to C57BL/6JJcl females for two generations. The heterozygous progeny were intercrossed to generate homozygous mutants (31 L and 100P) and wild-type control mice for behavioral testing.

### Animals and experimental design

Male 14-16 homozygous mutants and 14-16 wild-type control mice in each strain were used. The mice were transferred to Fujita Health University from the National Museum of Emerging Science and Innovation at the age of 7-14 weeks. They were group-housed (two mutant and two control mice per cage) in a plastic cage (22.7 × 32.3 × 12.7 cm) after weaning in a room with a 12-hr light/dark cycle (lights on at 7:00 am) with access to food and water ad libitum. Room temperature was kept at 23 ± 2°C. After acclimating for more than 2 weeks, behavioral testing was performed between 9:00 am and 6:00 pm. After the tests, all apparatus were cleaned with diluted sodium hypochlorite solution to prevent a bias due to olfactory cues. Experiments were conducted in the following sequence: the neurological screen and neuromuscular strength test, light/dark transition test, open field test, elevated plus maze test, hot plate test, one-chamber social interaction test, rotarod test, startle response/prepulse inhibition test, Porsolt forced swim test, Crawley's sociability and social novelty preference test, T-maze test, tail suspension test, and the contextual and cued fear conditioning test (Table 1). Behavioral tests were performed at intervals of at least 1 day. After Crawley's sociability and social novelty preference test, one 31 L mouse died, and 15 mutants were used for the subsequent tests. Three 31 L mutants and the one control mouse were observed to be injured on their back and they were singly housed one day before or after the beginning of food restriction for T-maze test. Since exclusion of the data obtained from the four singly-housed mice yielded essentially identical statistical results in any behavioral tests conducted after the isolation, we included the data taken from the four mice in our study. During the tail suspension test, because one mutant mouse fell off the apparatus, the data of 14 mutants were analyzed. For 100P mice, during the elevated plus maze test, one control mouse fell off the apparatus, and the subject's data were excluded from analysis. For day 1 of the Porsolt forced swim test, the data of one control mouse were excluded because of drowning. 31 L mutants and the wild-type control mice were tested between September 27, 2010 and February 2, 2011, and 100P mutants and the wild-type control mice were tested between February 21, 2011 and May 19, 2011. In each strain, the mutants and the control mice were tested at the same time in any behavioral tests. The behavioral data will be disclosed as part of a public database http://www.mouse-phenotype.org/, and users will be able to view the database after registration. All behavioral testing procedures were approved by the Institutional Animal Care and Use Committee of Fujita Health University.

**Table 1:** Comprehensive behavioral test battery of Disc1 mutant mice

Test	Q31L		L100P		
	
	Age (w)	Days	Age (w)	Days	Results
1.Neurological screen	10-17	1-2	11-15	1-3	Figure 1A-D
2.Light/dark transition	11-17	4	12-15	4	Figure 2I-L
3.Open field	11-18	7-9	12-16	8-10	Figure 2A-H
4.Elevated plus maze	12-18	11-12	13-16	11-12	Figure 2M-P
5.Hot plate	12-19	15	13-16	14	Figure 3A
6.Social Interaction	13-19	16	13-17	15	Figure 4A-E
7.Rotarod	13-19	18-19	13-17	16-17	Figure 3B, C
8.Prepulse inhibition	13-20	21	16-19	33	Figure 5E, F
9.Porsolt forced swim	14-20	24-25	17-20	39-40	Figure 5A, B
10.Socialbility/social novelty	15-21	30-35	17-21	43-46	Figure 4F, G
11.T-maze	24-30	94-113	18-22	51-75	Figure 6A-D
12.Tail suspension	27-34	120	23-26	81	Figure 5 C, D
13.Fear conditioning	29-35	128-129	23-27	87-88	Figure 6E-J

Age (w): age of weeks of subjects at the beginning of each test

### Neurological screen and neuromuscular strength

Neurological screen and neuromuscular strength tests were performed as previously described [28]. The righting, whisker touch, and ear twitch reflexes were evaluated. Physical features, including the presence of whiskers or bald hair patches, were also recorded. A grip strength meter (O'HARA & Co., Tokyo, Japan) was used to assess forelimb grip strength. Mice were lifted by holding the tail so that their forepaws could grasp a wire grid. The mice were then gently pulled backward by the tail with their posture parallel to the surface of the table until they released the grid. The peak force applied by the forelimbs of the mouse was recorded in Newtons (N). Each mouse was tested three times, and the greatest value measured was used for data analysis. In the wire hang test, the mouse was placed on a wire mesh that was then inverted slowly, so that the mouse gripped the wire not to fall off. Latency to fall was recorded, with a 60 s cut-off time.

### Light/dark transition test

A light/dark transition test was conducted as previously described [29]. The apparatus was consisted of a cage (21 × 42 × 25 cm) divided into two sections of equal size by a partition with a door (O'HARA & Co., Tokyo, Japan). One chamber was brightly illuminated (390 lux), whereas the other chamber was dark (2 lux). Mice were placed into the dark chamber and allowed to move freely between the two chambers with the door open for 10 min. The total number of transitions, latency to first enter the lit chamber, distance traveled, and time spent in each chamber were recorded by Image LD software (see 'Data analysis').

### Open field test

Each mouse was placed in the corner of the open field apparatus (40 × 40 × 30 cm; Accuscan Instruments, Columbus, OH, USA). The apparatus was illuminated at 100 lux. Total distance traveled (in cm), vertical activity (rearing measured by counting the number of photobeam interruptions), time spent in the center area, and beam-break counts for stereotyped behaviors were recorded. Center area was defined as 1 cm away from the walls. Data were collected for 120 min.

### Elevated plus maze test

An elevated plus maze test was conducted as previously described [30]. The elevated plus maze consisted of two open arms (25 × 5 cm) and two enclosed arms of the same size with 15-cm high transparent walls, which arms were connected by a central square (5 × 5 cm) (O'HARA & Co., Tokyo, Japan). The open arms were surround by a raised ledge (3-mm thick and 3-mm high) to avoid mice falling off the arms. The arms and central square were made of white plastic plates and were elevated 55 cm above the floor. Arms of the same type were located opposite from each other. Each mouse was placed in the central square of the maze (5 × 5 cm), facing one of the closed arms. The number of entries into open arms and the time spent in the open or enclosed arms were recorded during a 10-min test period. Percentage of entries into open arms, time spent in open arms (s), number of total entries, and total distance traveled (cm) were calculated. Data acquisition and analysis were performed automatically, using Image EP software (see 'Data analysis').

### Hot plate test

The hot plate test was used to evaluate sensitivity to a painful stimulus. Mice were placed on a 55.0 (± 0.1)°C hot plate (Columbus Instruments, OH, USA), and latency to the first fore- or hind-paw response was recorded. The paw response was defined as either a paw lick or a foot shake.

### Social interaction test in a novel environment

In the social interaction test, two mice of identical genotypes that were previously housed in different cages were placed in a box together (40 × 40 × 30 cm) (O'HARA & Co., Tokyo, Japan) and allowed to explore freely for 10 min [27]. Behavior was recorded and analyzed automatically using Image SI program (see 'Data analysis'). The total number of contacts, total duration of active contacts, total contact duration, mean duration per contact, and total distance traveled were measured. The active contact was defined as follows. Images were captured at 1 frame per second, and distance traveled between two successive frames was calculated for each mouse. If the two mice contacted each other and the distance traveled by either mouse was longer than 10 cm, the behavior was considered as an 'active contact'.

### Rotarod test

Motor coordination and balance were tested with the rotarod test. The rotarod test, using an accelerating rotarod (UGO Basile Accelerating Rotarod), was performed by placing mice on rotating drums (3 cm diameter) and measuring the time each animal was able to maintain its balance on the rod. The speed of the rotarod accelerated from 4 to 40 rpm over a 5-min period.

### Startle response/prepulse inhibition test

A startle reflex measurement system (O'HARA & Co., Tokyo, Japan) was used to measure startle response and prepulse inhibition. A test session began by placing a mouse in a plastic cylinder where it was left undisturbed for 10 min. White noise (40 ms) was used as the startle stimulus for all trial types. The startle response was recorded for 140 ms (measuring the response every 1 ms) starting with the onset of the prepulse stimulus. The background noise level in each chamber was 70 dB. The peak startle amplitude recorded during the 140 ms sampling window was used as the dependent variable. A test session consisted of six trial types (i.e., two types for startle stimulus only trials, and four types for prepulse inhibition trials). The intensity of the startle stimulus was 110 or 120 dB. The prepulse sound was presented 100 ms before the startle stimulus, and its intensity was 74 or 78 dB. Four combinations of prepulse and startle stimuli were used (74-110, 78-110, 74-120, and 78-120 dB). Six blocks of the six trial types were presented in pseudorandom order such that each trial type was presented once within a block. The average inter-trial interval was 15 s (range 10-20 s).

### Porsolt forced swim test

A transparent plastic cylinder (20 cm height × 10 cm diameter) filled with water (21-23°C) up to a height of 7.5 cm was put in a white plastic chamber (31 × 41 × 41 cm) (O'HARA & Co., Tokyo, Japan). Mouse was placed into the cylinder, and the immobility and the distance travelled were recorded over a 10-min test period. Images were captured at one frame per second. For each pair of successive frames, the amount of area (pixels) within which the mouse moved was measured. When the amount of area was below a certain threshold, mouse behavior was judged as "immobile." When the amount of area equaled or exceeded the threshold, the mouse was considered as "moving." The optimal threshold by which to judge was determined by adjusting it to the amount of immobility measured by human observation. Immobility lasting for less than a 2 sec. was not included in the analysis. Data acquisition and analysis were performed automatically, using Image PS software (see 'Data Analysis').

### Crawley's sociability and social novelty preference test

The testing apparatus consisted of a rectangular, three-chambered box and a lid with an infrared video camera (O'HARA & Co., Tokyo, Japan). Each chamber was 20 × 40 × 22 cm and the dividing walls were made from clear Plexiglas, with small square openings (5 × 3 cm) allowing access into each chamber. An unfamiliar C57BL/6 J male (stranger 1), that had had no prior contact with the subject mice, was placed in one of the side chambers. The location of stranger 1 in the left vs. right side chamber was systematically alternated between trials. The stranger mouse was enclosed in a small, round wire cage, which allowed nose contact between the bars, but prevented fighting. The cage was 11 cm in height, with a bottom diameter of 9 cm, vertical bars 0.5 cm apart. The subject mouse was first placed in the middle chamber and allowed to explore the entire test box for a 10-min session. The amount of time spent in each chamber was measured with the aid of a camera fitted on top of the box. Each mouse was tested in a 10-min session to quantify social preference for the first stranger. After the first 10-min session, a second unfamiliar mouse was placed in the chamber that had been empty during the first 10-min session. This second stranger was also enclosed in an identical small wire cage. The test mouse thus had a choice between the first, already-investigated unfamiliar mouse (stranger 1), and the novel unfamiliar mouse (stranger 2). The amount of time spent in each chamber during the second 10-min was measured as described above. Data acquisition and analysis were performed automatically using Image J based original program (see 'Data Analysis').

### T-maze test

The forced alternation task was conducted using an automatic T-maze apparatus (O'HARA & Co., Tokyo, Japan). It was constructed of white plastics runways with walls 25-cm high. The maze was partitioned off into 6 areas by sliding doors that can be opened downward. The stem of T was composed of area S2 (13 × 24 cm) and the arms of T were composed of area A1 and A2 (11.5 × 20.5 cm). Area P1 and P2 were the connecting passage way from the arm (area A1 or A2) to the start compartment (area S1). The end of each arm was equipped with a pellet dispenser that could provide food reward. The pellet sensors were able to record automatically pellet intake by the mice. One week before the pre-training, mice were deprived of food until their body weight was reduced to 80-85% of the initial level. Mice were kept on a maintenance diet throughout the course of all the T-maze experiments. Before the first trial, mice were subjected to 30-min adaptation sessions, during which they were allowed to freely explore the T-maze with all doors open and both arms baited with food. On the day after the adaptation session, mice were subjected to a forced alternation protocol for 8 days (one session consisting of 10 trials per day; cut-off time, 50 min). Mice were given 10 pairs of training trials per day. On the first (sample) trial of each pair, the mouse was forced to choose one of the arms of the T (area A1 or A2), and received the reward at the end of the arm. After the mouse consumed the pellet or the mouse stayed more than 30 sec without consuming the pellet, door that separated the arm (area A1 or A2) and connecting passage way (area P1 or P2) would be opened and the mouse could return to the starting compartment (area S1), via connecting passage way, by itself. In this way, the potential stress could be reduced compared to the traditional forced alternation paradigm in which human experimenter brings back the mouse to the start box by hand. The mouse was then given 3 sec delay there and a free choice between both T arms and rewarded for choosing the other arm that was not chosen on the first trial of the pair. Choosing the incorrect arm resulted in no reward and confinement to the arm for 10 sec. The location of the sample arm (left or right) was varied pseudo-randomly across trials using Gellermann schedule so that mice received equal numbers of left and right presentations. A variety of fixed extra-maze clues surrounded the apparatus. On the 6-8th day, delay (10, 30 or 60 sec) was applied after the sample trial. Data acquisition, control of sliding doors, and data analysis were performed by Image TM software (see 'Image analysis').

### Tail suspension test

The tail suspension test was performed for a 10-min test session. Mice were suspended 30 cm above the floor of a white plastic chamber (31 × 41 × 41 cm) (O'HARA & Co., Tokyo, Japan) in a visually isolated area by adhesive tape placed ~1 cm from the tip of the tail, and the behavior was recorded over a 10-min test period. Images were captured at one frame per second. As similar in Porsolt forced swim test, immobility was judged by the application program according to a certain threshold. Immobility lasting for less than a 2 sec. was not included in the analysis. Data acquisition and analysis were performed automatically, using Image TS software (see Section 'Data analysis').

### Contextual and cued fear conditioning test

Each mouse was placed in a transparent acrylic chamber (33 × 25 × 28 cm) with a stainless-steel grid floor (0.2 cm-diameter, spaced 0.5 cm apart) (O'HARA & Co., Tokyo, Japan) illuminated at 100 lux and allowed to explore freely for 2 min. A 55 dB white noise, which served as the conditioned stimulus (CS), was presented for 30 sec, followed by a mild (2 sec, 0.3 mA) footshock, which served as the unconditioned stimulus (US). Two more CS-US pairings were presented with a 2-min inter-stimulus interval. Context testing was conducted 24 h after conditioning in the same chamber. Cued testing with altered context was conducted after conditioning using a triangular box (33 × 29 × 32 cm) made of white opaque Plexiglas, which was located in a different room. The chamber of the test was illuminated at 30 lux. Data acquisition, control of stimuli (i.e. white noises and shocks), and data analysis were performed automatically, using Image FZ software (see 'Data analysis'). Images were captured at 1 frame per second. For each pair of successive frames, the amount of area (pixels) by which the mouse moved was measured. When this area was below a certain threshold (i.e., 30 pixels), the behavior was judged as 'freezing'. When the amount of area equaled or exceeded the threshold, the behavior was considered as 'non-freezing'. The optimal threshold (amount of pixels) to judge freezing was determined by adjusting it to the amount of freezing measured by human observation. 'Freezing' that lasted less than the defined time threshold (i.e., 2 sec) was not included in the analysis. The parameters were constant for all mice assessed.

### Data analysis

Behavioral data were obtained automatically through applications based on the public domain NIH Image program and the ImageJ program, and they were modified for each test by Tsuyoshi Miyakawa (available through OHARA & Co., Tokyo, Japan). Statistical analysis was conducted using StatView (SAS Institute, Cary, NC, USA). Data were analyzed using two-tailed t-tests, two-way ANOVAs, or two-way repeated measures ANOVAs. Values in graphs are expressed as mean ± SEM.

## Results

### 1. Physical characteristics and neurological screen

**Figure 1:**
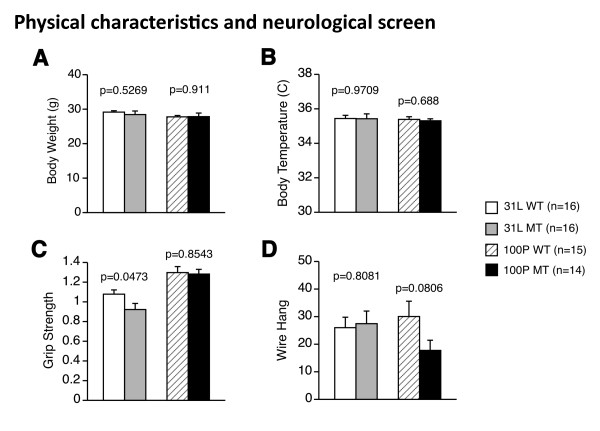
**General health and neurological screen**. (A) Body weight (g), (B) body temperature, (C) grip strength score, and (D) wire hang latency were recorded. 31 L and 100P mutant mice showed normal physical characteristics, except 31 L mutant mice had a lower grip strength score compared with wild-type control mice. Data represent the mean ± SEM. The p values indicate a genotype effect in a *t*-test.

31 L and 100P mutant mice appeared healthy and showed no obvious differences in physical characteristics relative to wild-type mice (body weight in 31 L, t(30) = 0.64, p = 0.5269; body weight in 100P, t(27) = 0.113, p = 0.911; body temperature in 31 L, t(30) = 0.037, p = 0.9709; body temperature in 100P, t(27) = 0.406, p = 0.688). However, 31 L mutant mice showed slightly but significantly less grip strength than wild-type mice (t(30) = 2.068, p = 0.0473). However, multiple statistical tests were conducted with our test battery strategy and the difference may not be considered as statistically significant after Bonferroni correction. Replication of the results would be needed to make a definitive conclusion about grip strength of the mutants. In the wire hang test, there was no significant difference in neuromuscular strength between the mutants and wild-type mice (t(30) = 0.245, p = 0.8081) (Figure 1). For 100P mutant mice, no significant differences in grip strength score (t(27) = 0.185, p = 0.8543) or wire hang latency (t(27) = 1.815, p = 0.0806) were found compared with wild-type mice.

### 2. Locomotor activity and anxiety-like behavior

**Figure 2:**
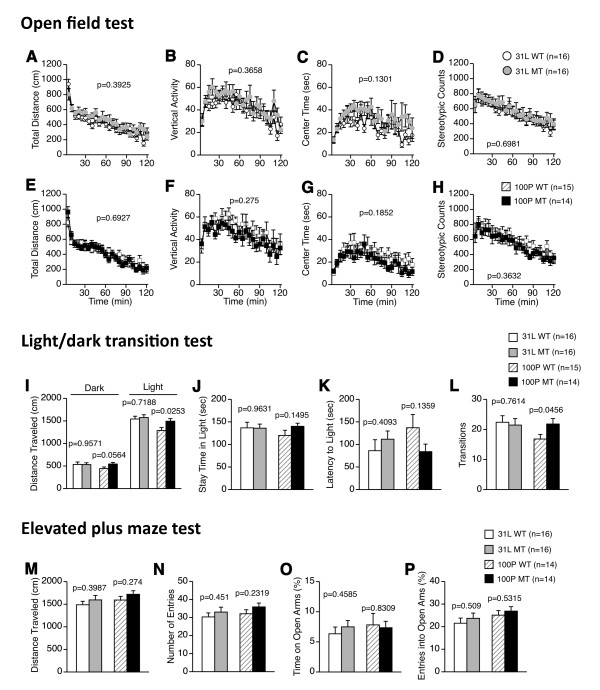
**Locomotor activity and anxiety-like behavior**. (A-H) Open field test: (A, E) total distance traveled, (B, F) vertical activity, (C, G) time spent in the center area, and (D, H) stereotypic counts were recorded. In each strain, there were no significant differences in the behavioral indices of the open field test between the genotypes. (I-L) Light/dark transition test: (I) distance traveled in the light/dark compartments, (J) time spent in the light compartment, (K) latency to enter the light compartment, and (L) number of light/dark transitions were recorded. 100P mutants traveled a higher distance in the light compartment and showed a higher number of transitions compared with the wild-type mice. (M-P) Elevated plus maze test: (M) distance traveled, (N) number of arm entries, (O) percentage of time spent in open arms, and (P) percentage of entries into the open arms were calculated. No genotype effects were found in this test. Data represent the mean ± SEM. The p values indicate a genotype effect in a *t*-test.

In the open field test (Figure 2A-H), there were no significant main effects of genotype on total distance, vertical activity, center time, and stereotypic counts in 31 L mouse line (F(1, 30) = 0.753, p = 0.3925; F(1, 30) = 0.843, p = 0.3658; F(1, 30) = 2.423, p = 0.1301; F(1, 30) = 0.153, p = 0.6981, respectively) or in 100P mouse line (F(1, 27) = 0.16, p = 0.6927; F(1, 27) = 1.242, p = 0.275; F(1, 27) = 1.849, p = 0.1852; F(1, 27) = 0.855, p = 0.3632, respectively). Also, there were no significant genotype × time interactions for any indices of the open field test.

For the light/dark transition test, in the 31 L mouse line, there were no significant differences in the distance traveled in the light/dark compartments, time spent in the light compartment, latency to enter the light compartment, or the number of light/dark transitions between the mutant and wild-type mice (t(30) = 0.054, p = 0.9571; t(30) = 0.363, t = 0.7188; t(30) = 0.047, p = 0.9631; t(30) = 0.837, p = 0.4093; t(30) = 0.306, p = 0.7614, respectively) (Figure 2I-L). In the 100P mouse line, the time spent in the light chamber and the first latency to enter the light chamber did not differ significantly between genotypes (t(27) = 1.484, p = 0.1495; t(27) = 1.537, p = 0.1359, respectively), but the mutant mice showed a longer distance traveled (in the light chamber: t(27) = 1.994, p = 0.0564; in the dark chamber: t(27) = 2.369, p = 0.0253) and an increased number of transitions between the chambers (t(27) = 2.096, p = 0.0456) compared with the wild-type mice (Figure 2I-L). Although these apparent differences in locomotor activity do not survive Bonferroni correction, given the hyperlocomotor activity of 100P mutant mice reported by Clapcote et al. [9], it is likely that the mutants have a hyperactive phenotype under some circumstances.

In the elevated plus maze test, there were no significant main effects of genotype on any of the indices, including the distance traveled, number of arm entries, percentage of time spent in open arms, and the percentage of entries into the open arms, in any of the strains of mice (for 31 L, t(30) = 0.856, p = 0.3987; t(30) = 0.764, p = 0.451; t(30) = 0.751, p = 0.4585; t(30) = 0.668, p = 0.509, respectively: for 100P, t(26) = 1.117, p = 0.274; t(26) = 1.224, p = 0.2319; t(26) = 0.216, p = 0.8309; t(26) = 0.634, p = 0.5315, respectively) (Figure 2M-P).

### 3. Normal pain sensitivity and motor coordination/motor learning

**Figure 3:**
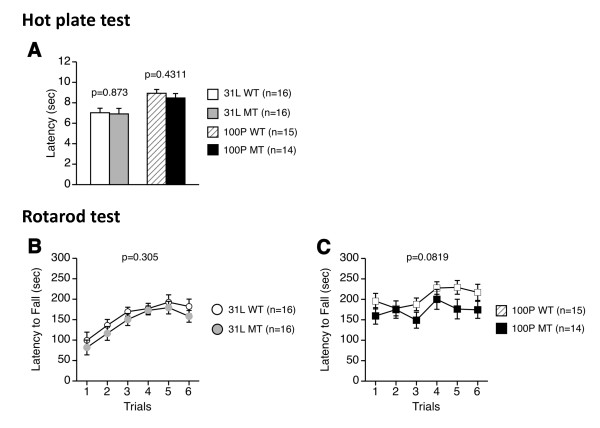
**Pain sensitivity and motor coordination/motor learning**. (A) Latency (sec) to the first fore- or hind-paw response in the hot plate test was measured. The latencies of 31 L and 100P mutant mice were similar to those of wild-type mice. (B, C) Latency (sec) to fall in the rotarod test was recorded. In each strain, there were no significant differences in the latency to fall between mutant and wild-type mice. Data represent the mean ± SEM. The p values indicate a genotype effect in a *t*-test (A) or a two-way repeated measures ANOVA (B, C).

To assess pain sensitivity and motor coordination/motor learning, 31 L and 100P mutant mice and wild-type mice were subjected to the hot plate test and the rotarod test. There were no significant effects of genotype on the latency to the first fore- or hind-paw response in the hot plate test (31 L, F(1, 30) = 1.089, p = 0.305; 100P, F(1, 27) = 3.267, p = 0.0819) (Figure 3A). Also, there were no significant main effects of genotype (31 L, F(5, 150) = 0.159, p = 0.9768; 100P, F(5, 135) = 0.574, p = 0.7202) and no significant genotype × trial interactions (31 L, F(5, 150) = 0.159, p = 0.9768; 100P, F(5, 135) = 0.574, p = 0.7202) for the latency to fall in the rotarod test (Figure 3B, C).

### 4. Normal sociability and social novelty preference

**Figure 4:**
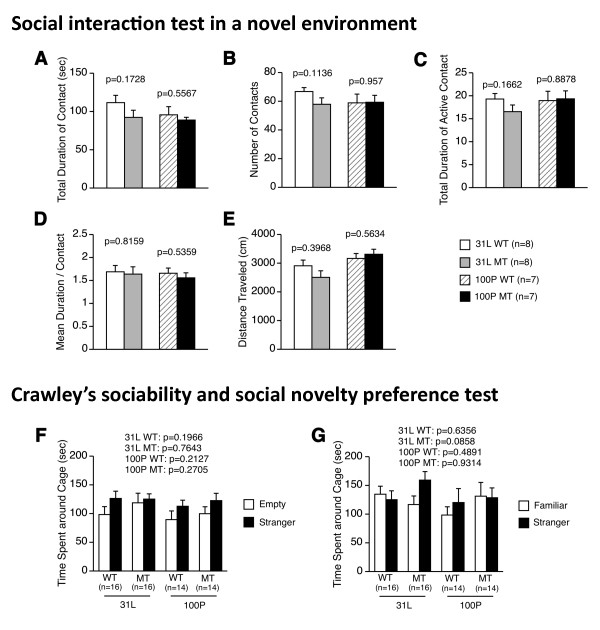
**Sociability and social novelty preference**. (A-E) Social interaction test in a novel environment: (A) total duration of contacts, (B) number of contacts, (C) total duration of active contacts, (D) mean duration of each contact, and (E) total distance traveled were analyzed. (F, G) Crawley's sociability and social novelty preference test: (F) time spent around the cage with stranger 1 or around the empty cage, and (G) time spent around the cage with stranger 1 and stranger 2 were measured. No significant differences in the behavioral indices between mutant and wild-type mice were found in each strain. Data represent the mean ± SEM. The p values indicate a genotype effect in a *t*-test.

In the social interaction test conducted in a novel environment (Figure 4A-E), there were no significant differences between the mutants and controls in any of the mouse strains in their total duration of contacts, number of contacts, total duration of active contacts, mean duration of contacts, or distance traveled (for 31 L, t(14) = 1.437, p = 0.1728; t(14) = 1.688, p = 0.1136; t(14) = 1.461, p = 0.1662; t(14) = 0.237, p = 0.8159; t(14) = 0.874, p = 0.3968, respectively: for 100P, t(12) = 0.605, p = 0.5567; t(12) = 0.055, p = 0.957; t(12) = 0.144, p = 0.8878; t(12) = 0.637, p = 0.5359; t(12) = 0.594, p = 0.5634, respectively).

For Crawley's sociability and social novelty preference test (Figure 4F, G), no significant differences were found between the duration of time spent around cage with stranger 1 and the duration of time spent around the opposite cage ("empty cage") in 31 L mutants and the controls (t(15) = 0.305, p = 0.7643; t(15) = 1.351, p = 0.1966, respectively) and in 100P mutants and the controls (t(13) = 1.151, p = 0.2705; t(13) = 1.31, p = 0.2127, respectively). Likewise, there were no significant differences between the durations of time spent around the cage with stranger 2 and stranger 1 in 31 L mutants and the controls (t(15) = 1.839, p = 0.0858; t(15) = 0.484, p = 0.6356, respectively) and in 100P mutants and the controls (t(13) = 0.088, p = 0.9314; t(13) = 0.712, p = 0.4891, respectively). These results indicate that sociability and social novelty preference did not significantly differ between the genotypes in 31 L and 100P mouse lines.

### 5. Normal depression-like behavior and sensorimotor gating

**Figure 5:**
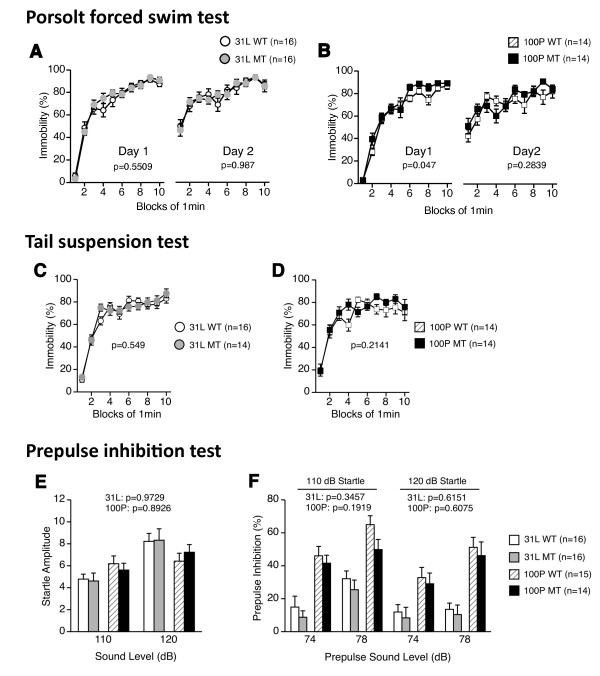
**Depression-like behavior and sensorimotor gating**. (A, B) Porsolt forced swim test: (A) immobility time (sec) on day 1 and day 2 in 31 L mutant and control mice and (B) in 100P mutant and control mice were recorded. On day 1, 100P mutants showed a significantly higher immobility time. (C, D) Tail suspension test: (C) immobility time (sec) in 31 L mutant and control mice and also (D) immobility time (sec) in 100P mutant and control mice were recorded. No significant differences in immobility time were found between the genotypes in each strain. (E, F) Startle response/prepulse inhibition test: (E) amplitudes of the startle response and (F) percentage of prepulse inhibition were recorded; there were no significant genotype effects on the behavioral indices in each strain. Data represent the mean ± SEM. The p values indicate a genotype effect in a *t*-test (A-D) or a two-way repeated measures ANOVA (E, F).

31 L and 100P mutant mice were investigated in the Porsolt forced swim test and the tail suspension test to assess depressive-like behavior. For the Porsolt forced swim test, in the 31 L mouse line, there were no significant effects of genotype on the percentage of immobility time for the habituation session on day 1 and for the test session on day 2 (F(1, 30) = 0.364, p = 0.5509; F(1, 30) = 0.0002, p = 0.987, respectively) (Figure 5A). In the 100P mouse line, the percentage of immobility time of mutant mice for the habituation session on day 1 was slightly greater than that of wild-type mice (genotype effect: F(1, 26) = 4.35, p = 0.047); there was no significant effect of genotype on the percentage of immobility time for the test session on day 2 (F(1, 26) = 1.197, p = 0.2839) (Figure 5B). In the tail suspension test (Figures 5C, D), no significant genotype effect was found for the percentage of immobility time in 31 L or 100P mouse lines (31 L, F(1, 28) = 0.368, p = 0.549; 100P, F(1, 26) = 1.622, p = 0.2141).

The 31 L and 100P mutant mice were further examined for sensorimotor gating in the prepulse inhibition test (Figure 5E, F, respectively). The amplitudes of the startle response and the percentage of prepulse inhibition (110 dB or 120 dB) in the mutants were similar to those of wild-type mice in 31 L mouse line (startle response, F(1, 30) = 0.001, p = 0.9729; 110 dB, F(1, 30) = 0.918, p = 0.3457; 120 dB, F(1, 30) = 0.258, p = 0.6151) and in 100P mouse line (startle response, F(1, 27) = 0.019, p = 0.8926; 110 dB, F(1, 27) = 1.792, p = 0.1919; 120 dB, F(1, 27) = 0.27, p = 0.6075). These results indicate that 31 L and 100P mutations do not induce any changes in depression-like behavior or sensorimotor gating.

### 6. Normal learning and memory functions

**Figure 6:**
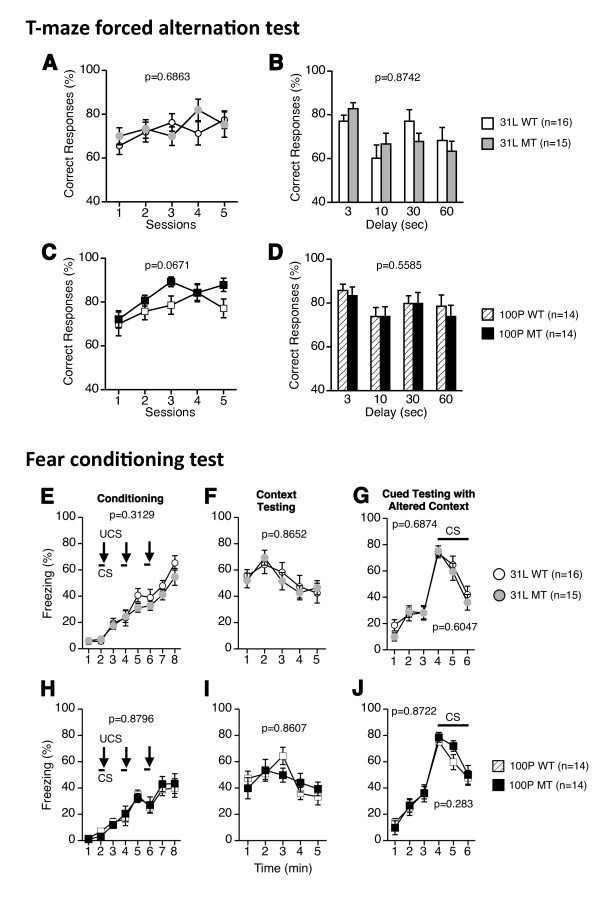
**Learning and memory**. (A-D) T-maze test (forced alternation task): (A, B) percent correct responses in the training session and delay session in 31 L mutant and control mice and (C, D) in 100P mutant and control mice were calculated. (E-J) Contextual and cued fear conditioning test: (E, H) the percentage of freezing time in conditioning, (F, I) in the context testing, and (G, J) in the cued test was recorded. In each strain, no significant genotype effects on freezing time were found in any of the tests. Data represent the mean ± SEM. Data were analyzed using two-way repeated measures ANOVAs and the p values indicate a genotype effect.

To evaluate working memory in 31 L and 100P mutant mice, their performance in the T-maze forced alternation task was investigated (Figure 6). The percentages of correct responses in 31 L and 100P mutant mice did not differ from those of wild-type control mice across sessions (F(1, 29) = 0.166, p = 0.6863; F(1, 26) = 3.652, p = 0.0671, respectively) (Figure 6A, C). Also, in the delayed alternation task, there was no significant difference in the percentage of correct responses between the mutant and wild-type mice in 31 L mouse line (t(30) = 0.856, p = 0.3987; t(30) = 0.764, p = 0.451; t(30) = 0.751, p = 0.4585; t(30) = 0.668, p = 0.509, respectively) or in 100P mouse line (t(26) = 1.117, p = 0.274; t(26) = 1.224, p = 0.2319; t(26) = 0.216, p = 0.8309; t(26) = 0.634, p = 0.5315, respectively) (Figure 6B, D). The results indicate that 31 L and 100P mutant mice did not show impaired working memory compared with wild-type control mice.

To further assess fear memory in 31 L and 100P mutant mice, the contextual and cued fear conditioning test was performed (Figure 6). No significant effects of genotype were found in the percentage of immobility time for conditioning (for 31 L, F(1, 29) = 1.055, p = 0.3129; for 100P, F(1, 26) = 0.023, p = 0.8796), context testing (for 31 L, F(1, 29) = 0.029, p = 0.8652; for 100P, F(1, 26) = 0.031, p = 0.8607), or altered context testing without or with cue presentation (for 31 L, F(1, 29) = 0.165, p = 0.6874; F(1, 29) = 0.274, p = 0.6047, respectively: for 100P, F(1, 26) = 0.026, p = 0.8722; F(1, 26) = 1.202, p = 0.283, respectively).

## Discussion

In the present study, we assessed the behavioral phenotypes of Disc1-Q31L and Disc1-L100P mutant mice with our comprehensive behavioral test battery. The results indicated that 31 L mutant mice did not show significant behavioral differences compared with wild-type control mice in any of the tests. Likewise, 100P mutant mice had similar phenotypes to wild-type control mice in almost all of the tests, except that 100P mutants traveled a longer distance in the light/dark transition test and were immobile slightly longer during the Porsolt forced swim test session on day 1. Thus, in our laboratory environment and using our experimental design, 100P mutant mice appeared to have some of the behavioral phenotypes relevant to symptoms of depression and schizophrenia, but neither mutant mouse strain showed any of the typical features in behavioral tests often used to assess the face validity of animal models of psychiatric disorders [31,32].

Our results were inconsistent with the previous findings that 31 L and 100P missense mutations are associated with depression-like behaviors and schizophrenia-like behaviors, respectively; 31 L mutant mice showed increased forced swim immobility, decreased social novelty preference, and deficits in prepulse inhibition and working memory, while 100P mutant mice displayed hyperactivity and deficits in prepulse inhibition, latent inhibition, and working memory [9]. There are several possible explanations for the inconsistency between our results and those reported by Clapcote et al. [9]. One possibility is that the two studies used mutant mouse lines with different genetic backgrounds. The ENU-induced mutant mouse lines were derived from the F1 progeny of C57BL/6 J and DBA/2 J mice, and therefore they were backcrossed to a C57BL/6 J strain to decrease the probability of the contribution of background genes to behavioral phenotypes. In Clapcote et al. [9], the mouse lines were backcrossed to N6 generations, and the genetic background of the mice was estimated to be 98.4% C57BL/6 J. We backcrossed the mouse lines to C57BL/6 J for two more generations, which presumably resulted in a background that was 99.6% C57BL/6 J. In addition, the original mouse lines could have had more heterozygous mutations occurring in regions flanking the mutation on the *Disc1 *gene. The founder *Disc1 *mutant was expected to be heterozygous for about 24 additional functional mutations randomly distributed across the approximately 25, 000 genes in the mouse genome, and the progeny of the sixth generation could be expected to have 0.75 additional heterozygous mutations, as discussed by Clapcote et al. [9]. In the present study, we estimated that the residual functional mutations of the mouse lines were reduced to 0.1875 heterozygous mutations by backcrossing for two more generations. The genetic background and flanking gene problems need to be considered in interpreting the results because genetic differences can potentially affect behavioral phenotypes in the mutant mouse lines [19,33].

The differences between the two studies' findings with regard to the behaviors of the mutant mice could also have arisen from differences in experimental variables and laboratory environments [34,35]. Indeed, there were differences in the methods of behavioral tests and experimental conditions between our study and the study of Clapcote et al. [9], such as differences in test apparatuses, test protocols, experimenter, age and sex of subjects, and prior test experiences of subjects. For example, although Clapcote et al. [9] conducted behavioral tests on sex-balanced groups of experimentally naïve mice at 12-16 weeks of age, we performed a test battery using male mice at 10-17 weeks of age at beginning of the test battery. Stressors are environmental factors that can affect the development of behavioral phenotypes relevant to psychiatric disorders [36-38]. In our laboratory environment, there may not have been sufficient stimuli to induce behavioral abnormalities in 31 L and 100P mutant mice.

There are several studies on mouse models with mutations in the *DISC1 *gene, such as mice with a 25-bp deletion in exon 6 of the *Disc1 *gene [11], transgenic mice expressing a dominant-negative form of *Disc1 *[10], transgenic mice with inducible expression of a *DISC1 *C-terminal fragment [12], and inducible forebrain-specific mutant *DISC1 *mice [13], which showed a range of behavioral abnormalities, including increased locomotor activity, working memory impairments, reduced social interaction, and a prepulse inhibition deficit. In the present study, we did not find typical behavioral phenotypes expected in animal models of psychiatric disorders in the mice with point mutations (31 L and 100P), although 100P mutant mice showed slightly increased activity. It is likely that the different behavioral phenotypes originated from the differences in the mutation methods of the *Disc1 *gene: a point mutation in our study, a 25-bp deletion [11], and inducible expression of dominant-negative form of *DISC1 *[10] or mutant human *DISC1 *[13]. Our study suggests that a point mutation, Q31L or L100P, on *Disc1 *gene has less functional impact on behavior than a gene knockdown or deletion does, although a possibility cannot be excluded that the discrepancies between our results and those from other studies might be due to the differences in other factors, such as genetic background, breeding and rearing conditions, test protocol, experimenter, and/or age and time at the testing. In any case, it will be needed to take our findings into consideration in using the ENU-induced *Disc1 *mutant mice as an animal model of psychiatric disorders.

In the present study, there were behavioral differences between the two control groups in several tests, although the two groups were genetically identical and bred under the same breeding environments in the same place. The behavioral differences could be due to the differences in breeding period in each strain, rearing condition, and/or age at the testing.

## Conclusion

The mouse lines generated by ENU mutagenesis are potentially useful tools for exploring the interplay among genes, the brain, and behavior. However, to clarify the precise functions of the gene, the genetic background of the mice must be carefully controlled prior to the behavioral experiment. In the present study, we backcrossed mutant mice to C57BL/6 J for an additional two generations, and we failed to reproduce previous results indicating that both 31 L and 100P mutant mice are animal models of depression and schizophrenia. Further behavioral studies under various experimental conditions may be necessary to confirm whether mice with these point mutations in the *Disc1 *gene are animal models of psychiatric disorders.

## Authors' contributions

TM was responsible for the original concept and the overall design of the research. YG and SW originally produced the 31 L and 100P mutant mouse lines and provided the two strains. YT and KT generated the mice for the behavioral tests. HS performed the behavioral tests and analyzed the data. HS and TM wrote the manuscript. All authors read and approved the final manuscript.

## Competing interests

The authors declare that they have no competing interests.
